# Supplementary material for: Evolution of Weyl orbit and quantum Hall effect in Dirac semimetal Cd3As2
Source: Nat Commun. 2017 Nov 2;8:1272. doi: 10.1038/s41467-017-01438-y (PMC5668429; doi:10.1038/s41467-017-01438-y)
Supplement: Supplementary file 1 — Supplementary Information [file 41467_2017_1438_MOESM1_ESM.pdf]

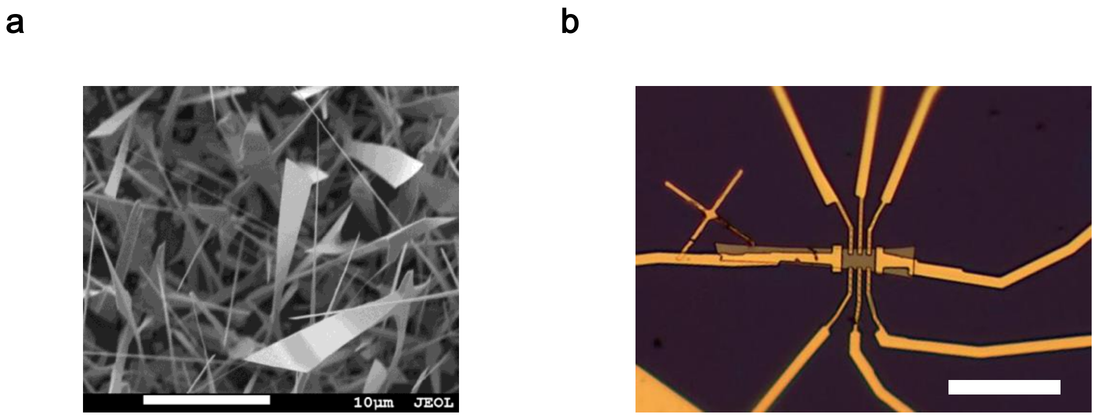

Supplementary Figure 1| Images of Cd_3As_2 nanoplates. (**a**) A typical scanning electron microscopy image of as-grown Cd_3As_2 nanoplates. The scale bar (white) in **a** is 10 μm. (**b**) An optical picture of Cd_3As_2 nanoplate device. The scale bar (white) in **b** is 50 μm.

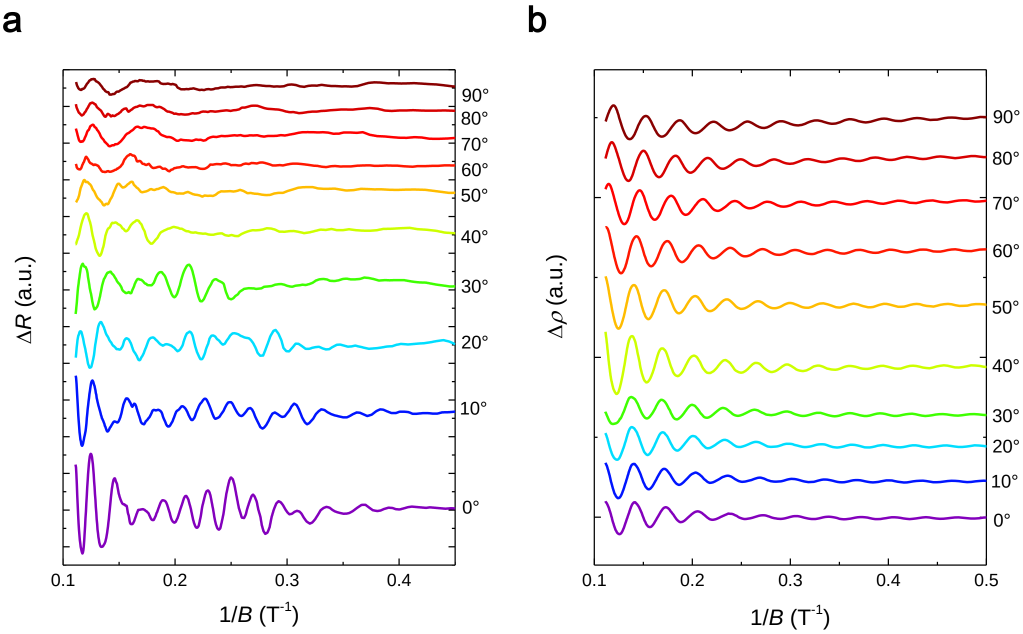

Supplementary Figure 2| SdH oscillations of Cd_3As_2 nanoplate and bulk crystal. The angle dependence of extracted SdH oscillations in sample #1 (**a**) and bulk Cd_3As_2 crystal (**b**) at 2.5 K. The angle θ is defined in the same way as Fig. 1b in the main text.

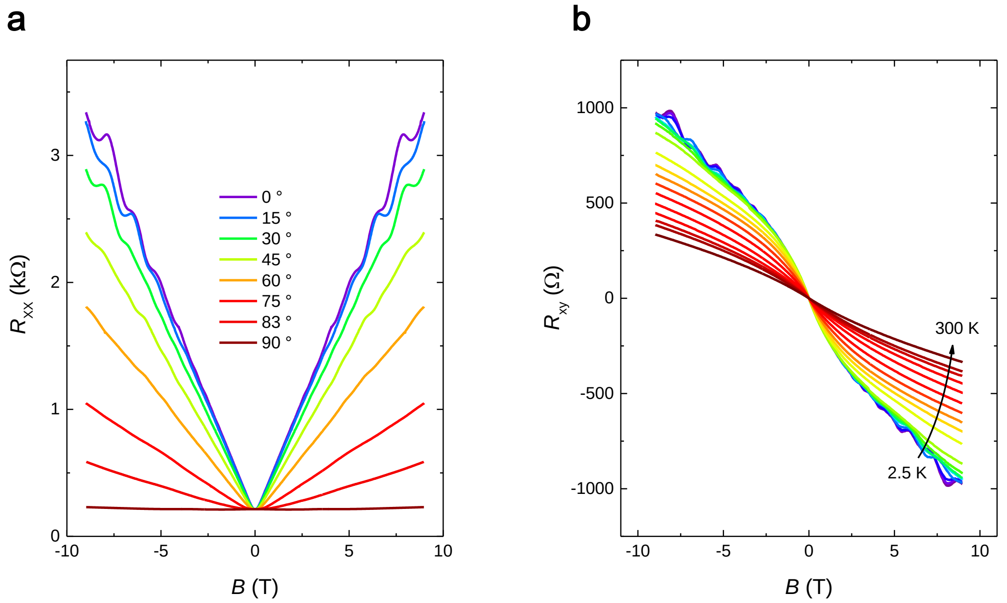

Supplementary Figure 3| Magnetotransport results of low-Fermi-level Cd_3As_2 nanoplates. (**a**) The field dependence of magnetoresistance R_{xx} of sample #2 at different angles at 2.5 K. (**b**) The Hall resistance R_{xy} of sample #2 at different temperatures.

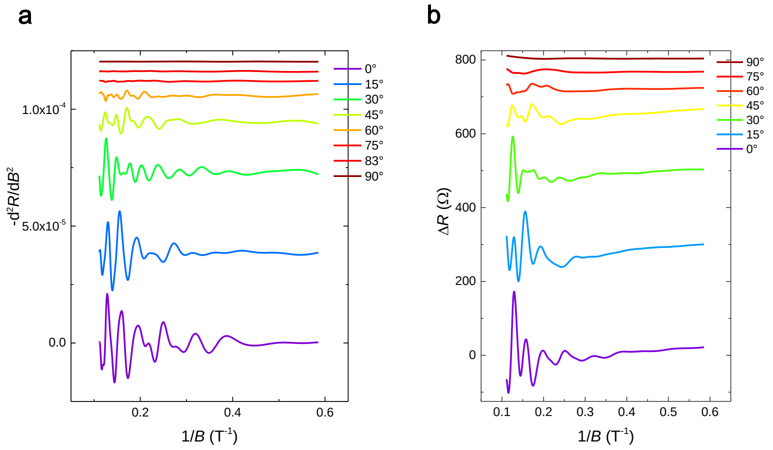

Supplementary Figure 4| Extracted quantum oscillations. (**a-b**) Comparison of ΔR (**a**) and $-\frac{d^2R}{dB^2}$ (**b**) curves in sample #2. The oscillation periodicity is obviously changed at high fields due to the splitting of the Landau levels, as shown in Fig. 2 in the main text.

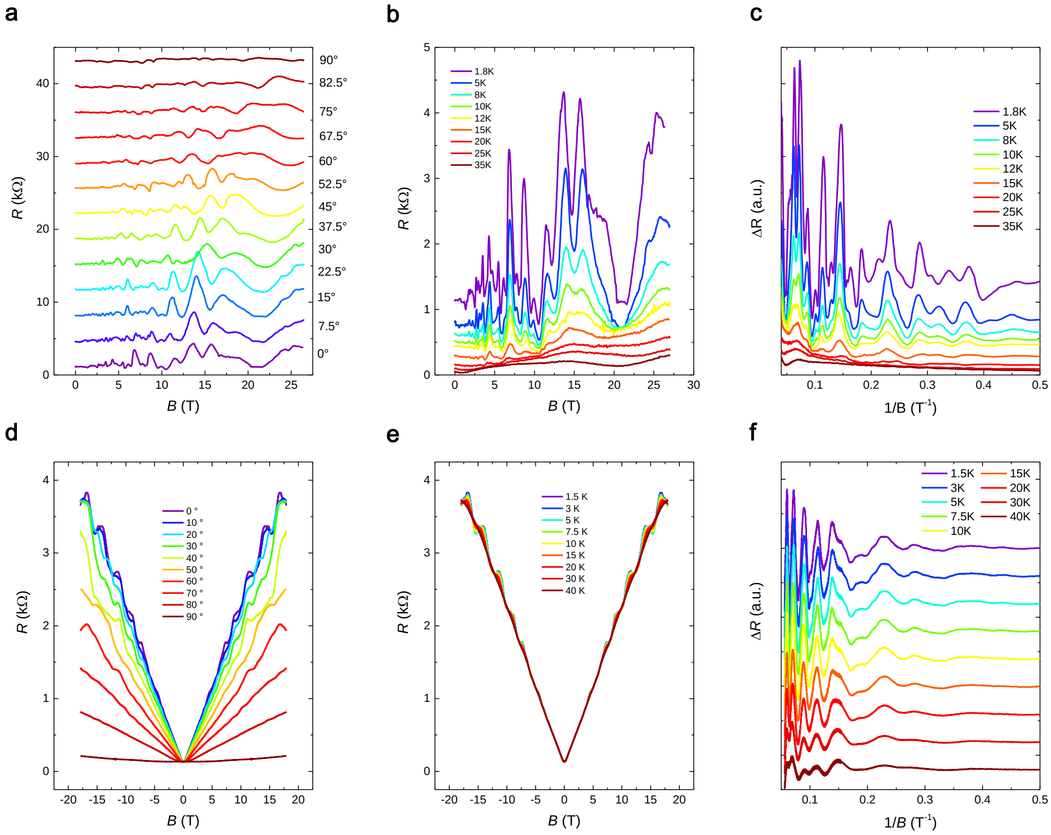

Supplementary Figure 5| Another two sets of magnetoresistance in Cd_3As_2 nanoplates. (a-c) The magnetoresistance R_{xx} of sample #4 at different angles (T=1.8 K) (**a**), different temperatures (θ=0°) (**b**), and oscillations plotted with 1/B at θ=0° (**c**). (**d-f**) The magnetoresistance R_{xx} of sample #5 at different angles (T=1.8 K) (**d**), different temperatures (θ=0°) (**e**), and oscillations plotted with 1/B at θ=0° (**f**).

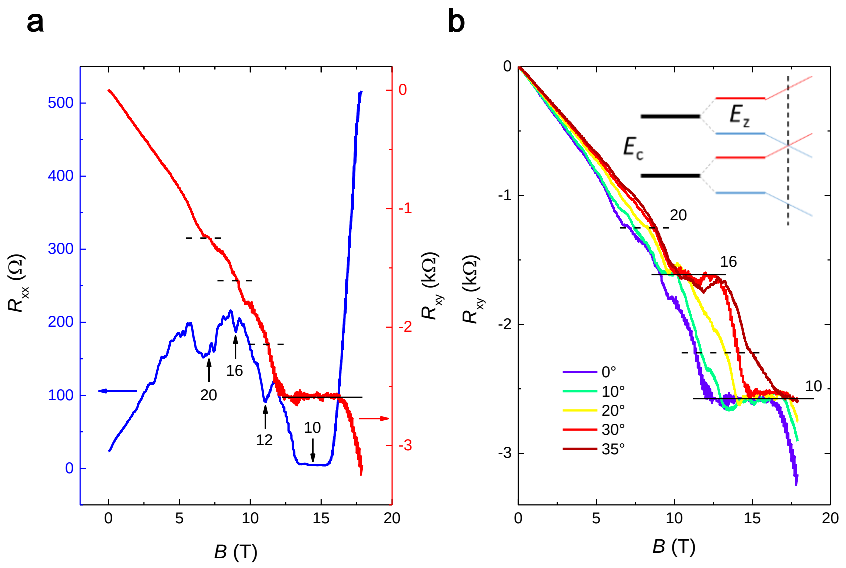

Supplementary Figure 6| Quantum Hall effect in Cd_3As_2 nanoplates. (**a**) Magnetic field dependence of magnetoresistance R_{xx} (blue) and Hall resistance R_{xy} (red) in sample #6. The filling factors are marked in the minima of R_{xx} based on the corresponding value of R_{xy}. (**b**) Hall resistance R_{xy} as the magnetic field is tilted away from out-of-plane direction (0°). The inset illustrates the overlapping of the splitted Landau levels as the Zeeman energy increases, which results in the dramatic change of filling factor from 16 to 10.

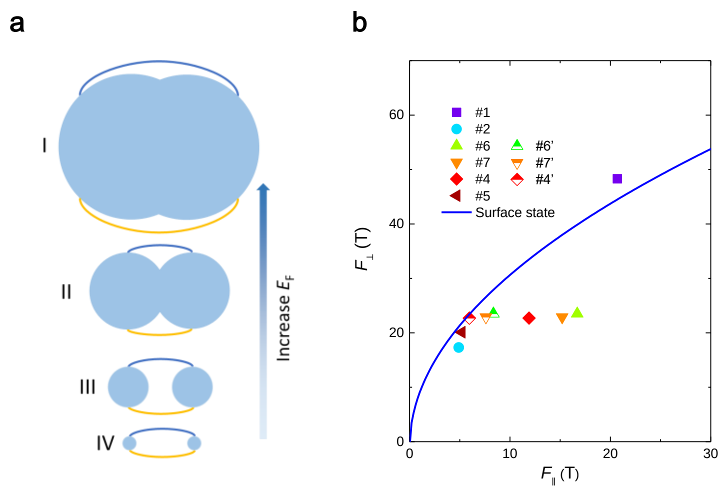

Supplementary Figure 7| Influence of the Lifshitz transition. (**a**) Schematic depiction of the Fermi surface with increasing bulk Fermi energy. (**b**) The relationship of the oscillation frequency $F_\perp$ with out-of-plane magnetic field (θ=0°) and $F_\parallel$ with in-plane magnetic field (θ=90°). The blue and red curves describe two predicted relationships between $F_\parallel$ and $F_\perp$ based on the surface-state and confined-bulk-state scenarios, respectively. Note that a corrected $F_\parallel$ in sample #4, #6 and #7 is displayed as marked by #4', #6' and #7', which is nearly half of the original value of $F_\parallel$ (marked by #4, #6 and #7) owing to the Lifshitz transition in bulk Fermi surface. The influence of Lifshitz transition will be gradually smeared out when the Fermi level continues increasing and the overlapped regime becomes dominating.

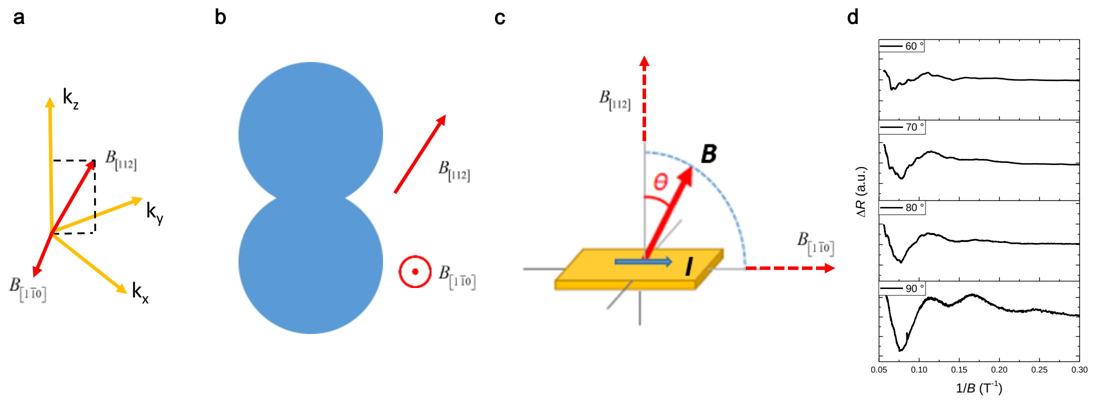

Supplementary Figure 8| Influence of bulk orbit when tilting magnetic field. (**a**) The field direction in momentum space. Note that the Weyl nodes of Cd_3As_2 are in k_z axis. (**b**) The projection view from the $(1\bar{1}0)$ plane. (**c**) Illustration of the field direction with respect to the device geometry. (**d**) Extracted quantum oscillation data in sample #6. The bulk oscillations are overwhelmed by the surface oscillations below 60 degree. The oscillation frequency is nearly unchanged in the regime of 60~90 degree.

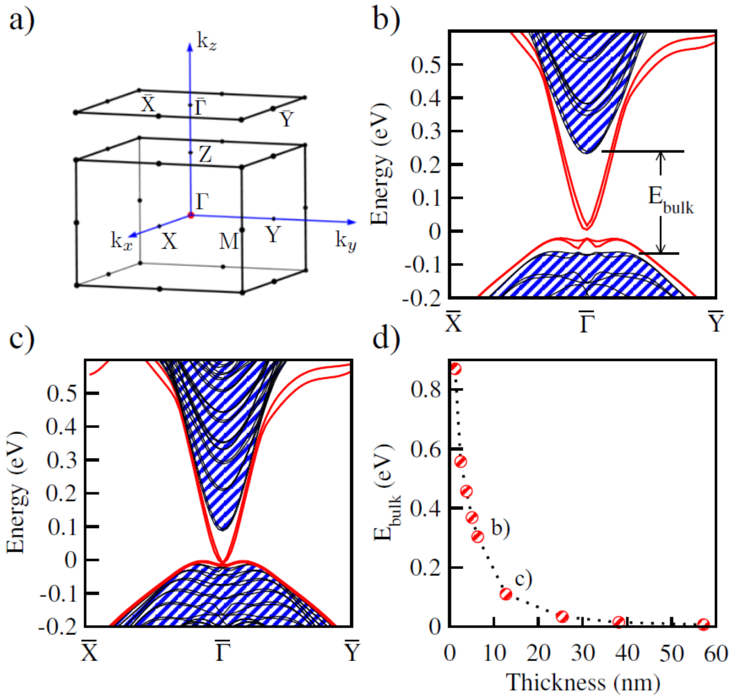

Supplementary Figure 9| Finite size effect of Cd_3As_2. (**a**) Brillouin zone for the P42/nmc unit cell used for the first-principles-derived tight-binding computations. Band structures for slabs with representative thicknesses (**b**) 8 nm and (**c**) 12 nm are shown, with the bulk bands shaded in blue. The surface bands are highlighted in red. (**d**) Variation of the bulk band gap, E_{bulk}, with increasing thickness of the slab. Note that here the confinement is added on the (001) direction for simplicity in calculation.

Sample	Thickness (nm)	F_B (T)	F_S (T)	Sheet carrier density (cm^{-2})
#1	~120	20.7	48.3	-2.3×10^{13}
#2	~100	4.9	17.3	-5.7×10^{12}
#4	~140	11.9	22.7	-1.5×10^{13}
#5	~110	5.2	20.1	-5.8×10^{12}
#6	~80	16.7	23.5	-3.5×10^{12}
#7	~80	15.2	22.9	-2.9×10^{12}
Moll *et al.*	/	36.5	61.5	/

Supplementary Table 1| Summary of sample parameters. F_B and F_S represent the bulk and surface oscillation frequency, respectively. The sheet carrier density is calculated from Hall coefficient. The last data set marked by Moll *et al.* is adopted from the previous literature[1].

Supplementary Note 1. Transport measurements of Cd_3As_2 nanostructures

Supplementary Figure 2a is the extracted Shubnikov–de Haas (SdH) oscillations in samples #1 at different angles. Clear multi-frequency oscillations and beating pattern can be observed at θ=0° while they disappear at θ=90°. As a comparison, SdH oscillations of another bulk sample are provided in Supplementary Fig. 2b, showing textbook-like single frequency oscillation at all angles, consistent with previous reports[2, 3]. Note that here the bulk Cd_3As_2 is also grown by the chemical vapor deposition method[4] with the similar growth conditions as sample #1. The typical thickness of the bulk samples is over 100 μm, well above the electron mean free path in Cd_3As_2 given by quantum oscillations[2]. Therefore, the Weyl orbit cannot be formed in this kind of bulk samples. The bulk frequencies in these two kinds of samples are close (Fig. 1d in the main text), indicating the similar Fermi levels. It helps to exclude the possibility of Fermi surface variation with different electron doping levels. Supplementary Figure 3 is the original magnetoresistance (**a**) and Hall resistance (**b**) of sample #2 at different angles. The field dependence of Hall resistance shows non-linear behavior possible due to a slight hole conduction since the Fermi level in sample #2 is close to Dirac points. Supplementary Figure 4 is a comparison of SdH oscillations revealed through the second derivative approach (**a**) and background subtraction (**b**), respectively. The second derivative method is more sensitive and gives more details about the oscillations. Supplementary Figure 5 displays the transport data of sample #4 and #5, both showing the change of oscillation interval at high magnetic field.

In the main text, we use the Landau fan diagram to extract the Fermi surface of sample #2. This method is very useful in dealing with single frequency oscillations. But for multiple frequency oscillations, one should be aware of the oscillation beating behavior, which will change the original peak positions. This is the reason why we did not use this method for sample #1. Here we argue that for sample #2, this method is still valid. The bulk oscillations only coexist with the surface oscillations at 45 and 60 degree. In other angles of sample #2, there is no obvious two-frequency-coexisting behavior except for the Zeeman splitting at high fields. For the oscillation data at these two angles, the surface frequency (36 T for 60 degree and 25 T for 45 degree) is several times larger than the bulk frequency (4.9 T). And their major oscillations take place in the different field regime (2~6 T for bulk oscillations and 5~9 T for surface oscillations). Meanwhile, since the bulk oscillation position does not change significantly with angle, we can use the large-angle data to help determine the bulk Landau level positions. Therefore, we believe that the method is applicable to sample #2.

Supplementary Figure 6a reveals the quantum Hall effect of the sample #6 with the marked filling factors for each Landau level (LL). One notable feature is that the Hall plateau directly jumps from $\nu = 16$ to $\nu = 10$ (Supplementary Fig. 6b). It originates from the overlapping of two splitted LLs as the field increases where the Zeeman energy E_z approaches the cyclotron energy E_c. For a fixed magnetic field, the energy gap of LL with a filling factor of $2n$ is given by $\Delta E_{\mathrm{even}} = E_{\mathrm{c}} - E_{\mathrm{z}} - \Gamma$ when n is an even number and $\Delta E_{\mathrm{odd}} = E_{\mathrm{z}} - \Gamma$ when n is an odd number.[5] Here Γ is the LL broadening factor caused by disorder and scattering. In our case, the original band of

$\nu = 8$ splits into two bands and one of them crosses the Fermi level along with the original band of $\nu = 12$ within one oscillation interval, giving rise to a change of 6 in the filling factor. Although a weak oscillation valley corresponding to $\nu = 12$ is witnessed in the longitudinal resistance R_{xx} at $\theta = 0°$, there is no well-defined plateau presented in the Hall resistance R_{xy} due to the relatively small energy gap between LLs.

Supplementary Table 1 summarizes a list of physical parameters in all the samples presented in this study. The sheet carrier density is calculated from Hall coefficient. The difference among samples mostly comes from the carrier density (or Fermi level). Such a change of Fermi level will affect the transport on the following aspects. Firstly, it leads to a crossover from the coexistence of bulk and surface SdH oscillations to surface-dominant transport under out-of-plane magnetic field (0 degree). In the low-Fermi-level samples, the Fermi wavelength of bulk electrons is gradually approaching the sample thickness. It results in a significant decrease in the bulk oscillation amplitude due to an enhanced boundary scattering while the two-dimensional surface state is much less affected. The bulk oscillation is twenty times smaller in amplitude than the surface oscillation at 0 degree and it can only be detected near 90 degree where the surface oscillation vanishes. Secondly, with the decrease of Fermi level in Cd_3As_2 nanoplates, the Landau level gap near the Fermi level significantly increases in the same field range. After overcoming the Landau level broadening effect from thermal activation and scattering, the quantum Hall effect can be observed when the Fermi level is low enough.

And also, we shall note that the small deviation of thickness among samples #1~7 does not affect the analysis of Fermi surface area. Theoretically speaking, the sample thickness affects the transport properties of Weyl orbit in two ways. Firstly, with the decrease of the thickness, the component of surface transport gets enhanced. This feature has already been observed by Moll *et al.*[1] through the evolution of peak amplitude in the quantum oscillation FFT spectra of Cd_3As_2 microstructures with different thickness. Secondly, the Lifshitz-Onsager quantization rule for Weyl orbit at out-of-plane magnetic field can be written as $\frac{1}{B_n} = \frac{e}{S_k}\left[2\pi(n+\gamma) - L_z\left(\mathbf{k}_w \cdot \hat{\mathbf{B}} + \frac{2E_F}{v_\parallel}\right)\right]$.[6] Here B_n, S_k, L_z, $\mathbf{k}_w$, E_F, and $v_\parallel$ are the field position corresponds to n-th Landau level, the k-space area enclosed by the two Fermi arcs combined, the sample thickness, the vector connecting two Weyl nodes, Fermi level and Fermi velocity parallel to magnetic field direction $\hat{\mathbf{B}}$, respectively. This bulk propagating process in the Weyl orbit contributes to an additional phase shift to the quantum oscillation. Signature of such thickness-sensitive quantum phase has been previously found in the comparison of two samples with difference geometries.[1] From this equation, we also can find the oscillation frequency (Fermi surface area of Weyl orbit) is only determined by the projected area of the top and bottom Fermi arcs.

Supplementary Note 2. The origin of Zeeman splitting in two scenarios

In the confined-bulk scenario, the field-induced splitting is from a conventional Zeeman effect of the bulk band. The Zeeman splitting of SdH oscillations in Cd_3As_2 has been observed in previous reports[2, 7]. However, the Zeeman splitting of SdH oscillations becomes very unconventional for surface states in topological semimetals. Theoretically, just as the surface states in topological insulators, the Fermi arcs in Dirac and Weyl semimetals are also spin-polarized.[8, 9] Under magnetic field, the Zeeman effect only contributes an additional energy shift instead of causing a Landau level splitting due to the lack of spin degeneracy.[10] For Dirac semimetals, although there are two degenerated Weyl orbits[11], they should adopt the same response to magnetic field due to the same energy shift for each half circle from Zeeman effect, which is guaranteed by the conservation of inversion symmetry similar to three-dimensional topological insulators[10, 12]. Hence, the magnetic field cannot split the SdH oscillations of Weyl orbits in Dirac semimetals if only considering the surface states. Nevertheless, the bulk Weyl nodes will also show Zeeman effect under magnetic field. In fact, due to the coupling with orbit, the Zeeman effect can lead to a complicate phase diagram in Dirac semimetals, depending on the field strength as well as the field direction.[13] The resultant bulk Zeeman effect will change the projected position of each Weyl node on the surface state, therefore changing the Fermi arc length. In this case, the double Fermi arcs are no longer identical, leading to the Zeeman-like splitting in the SdH oscillations. The intimate coupling between Fermi arc and Weyl node is the origin of the different response to external magnetic field in topological semimetals and inversion-symmetric topological insulators. The surface states of the latter cannot be splitted by magnetic field but instead acquire the same Zeeman energy value for both surfaces.[12] Also owing to this unique Zeeman effect in topological semimetals, the splitting behavior is slightly different from the Zeeman splitting of quantum oscillations in conventional systems[14].

Supplementary Note 3. Influence of Lifshitz transition

Supplementary Figure 7 shows the evolution of Fermi surface when increasing the bulk Fermi energy. The two Fermi spheres can join together and form a larger Fermi surface, accompanied by the Lifshitz transition. As the system approaches the Lifshitz transition, the size of bulk Fermi surface will experience an abrupt increase and get doubled upon the transition point (situation II). When further increasing the Fermi energy, the two Fermi spheres will overlap greatly and becomes a quasi-sphere shape again. Based on our analysis, the abnormal trend in sample #4, #6 and #7 in Fig. 5c is actually due to the influence of Lifshitz transition.

Meanwhile, the Lifshitz transition can also be detected through the angle dependence of bulk oscillations. The bulk oscillation frequency will experience a sudden doubling when the field is rotated from parallel all the way to perpendicular with the line connecting two Fermi sphere centers. For Cd_3As_2 nanoplates in our experiments with (112) crystal plane, the merged orbit from two joint Fermi spheres can be detected when the field is along the $[1\bar{1}0]$ direction parallel to the current direction. Supplementary Figure 8a illustrates the field direction in momentum space where the Weyl nodes of Cd_3As_2 are in k_z axis. Supplementary Figure 8b is the projection view on the $(1\bar{1}0)$ plane. As shown in Supplementary Fig. 8c, the $[112]$ and $[1\bar{1}0]$ directions correspond to 0 and 90 degree, respectively. In order to eliminate the merged orbit, one needs to tilt the field towards a small range around k_z direction. Note that k_z is close to the $[112]$ direction, the normal axis of the sample surface, where the bulk oscillation can no longer be detected for the low Fermi level samples below the Lifshitz transition. As shown in Supplementary Fig. 8d, the bulk oscillations of sample #6 can only be tracked up to 60 degree, where the surface oscillations already show up. However, in this range, there is no dramatic change of oscillation frequency. The most likely reason is that the angle is still not close to k_z axis enough. But further tilting the field towards the $[112]$ direction will largely enhance the surface oscillations and make the bulk state undetectable. This direct measurements of Lifshitz transition through bulk Fermi surface may be applicable to the bulky samples without the significant contribution from surface oscillations (thickness up to micron-scale) or Cd_3As_2 nanostructures with (100) or (010) plane.

On the other hand, the Lifshitz transition may cause some problems in the formation of Weyl orbit since the bulk chiral mode is not well defined above Lifshitz energy. From the previous experimental report[1], quantum oscillations of the Weyl orbit were observed in high-Fermi-level samples whose Fermi level (E_F=192 meV) is well above the Lifshitz energy of Cd_3As_2, which is no more than 20 meV as given by different theoretical calculations and experiments[13, 15, 16]. It suggests that Weyl orbits may persist even when the chiral Landau level is not well defined. In this case, one possible way to form a closed loop is through other gapped bulk channels, similar to the case when the double Fermi arcs get mixed[17]. Then a tunneling process will happen to overcome the energy gap. Nevertheless, this influence of Lifshitz transition on Weyl orbit requires more experimental investigations to clarify, especially regarding the role of bulk chiral mode.

Supplementary Note 4. Finite size effect in Cd_3As_2

To quantitatively evaluate the finite size effect, we carried out theoretical calculations based on a simplified model of Cd_3As_2 slab[18]. According to our calculation results, the finite size effect becomes very significant below 10 nm. The hybridization gap falls off with increasing thickness, and is very close to zero for a thin film of thickness ~ 60 nm. Since Cd_3As_2 has an inverted gap, apart from the bulk Dirac point, it also exhibits surface Dirac crossings when confined in a quantum well geometry. These surface cones are highlighted in red in Supplementary Fig. 9 b and c. Note that in contrast to the bulk cone, the surface crossing is already gapless for films as thin as ~12 nm. This behavior is similar to the case of Na_3Bi[18], the other putative three-dimensional Dirac semimetal. Therefore, the surface state should persist in thin layers of Cd_3As_2 down to a few nanometers, even though it is deformed into Fermi pockets rather than Fermi arcs.

Supplementary Note 5. Magnetic field effect on the Dirac semimetal state

The Dirac nodes in Dirac semimetals are actually overlapping Weyl nodes with opposite chirality.[7, 8] To preserve the overlapping Weyl nodes from annihilation, additional crystalline point-group symmetry is required in Dirac semimetals.[8] Therefore, the Dirac nodes always occur along the high symmetry directions in the momentum space. In the presence of perturbations to the crystal symmetry, a hybridization gap may be induced, resulting in a topological phase transition. In our study, such perturbation could be induced by the external magnetic field when it is not oriented along the four-fold rotational symmetry axis (which is true for field perpendicular to the (112) surface of Cd_3As_2). A qualitative understanding of the magnetic field evolution can be obtained by using a semi-classical analysis proposed by Potter *et al.*[11] Firstly considering the case for low magnetic field, which does not significantly break the rotational symmetry that protects the Dirac points, the oscillations for each of the Weyl orbits occur independently with a quantization condition given by $\epsilon_n = (n+\gamma)\frac{\pi v}{L+k_0 l_B^2}$. Here L is the thickness of the slab, $l_B = 1/\sqrt{eB}$ is the magnetic length, k_0 is the Fermi arc length, and v is the group velocity. In contrast, when the applied magnetic field breaks the rotational symmetry, the bulk Landau levels of the two degenerate Weyl points, which comprise the Dirac point, can hybridize and become gapped. This can be appreciated by considering a continuum Hamiltonian around the Dirac points, $H = H_1 + H_2$, with $H_1 = v(k_x\tau_x\sigma_z - k_y\tau_y + k_z\tau_z)$ and $H_2 = \alpha k_0(k_x^2 - k_y^2)\tau_x\sigma_x$. Here we have separated out the linear (H_1) and quadratic (H_2) terms in k. For a magnetic field applied in the y-z plane, $\mathbf{B} = B(\cos\theta\,\hat{z} + \sin\theta\,\hat{y})$, one can choose the lowering operator as $a = l_B(k_x + ik_y\cos\theta - k_z\sin\theta)/\sqrt{2}$. Considering H_2 as perturbation

to the linear-in-*k* Hamiltonian, H_1, it is possible to find the matrix elements of H_2 for the lowest Landau state ($\zeta = 0$) as $\langle \zeta = 0|H_2|\zeta = 0\rangle = \frac{1}{2l_B^2}\alpha k_0 \sin^2\theta\, \tau_x\sigma_x$.[11] From this we obtain the hybridization gap as $\Delta = \alpha k_0 eB \sin^2\theta$. If the Fermi energy lies within this gap, it is possible that the double Fermi arcs will be deformed into one closed Fermi pocket.

Another consequence of magnetic field is the breaking of time reversal symmetry.[19, 20] By considering the exchange couplings induced by the magnetic field, we can in general separate the field-dependent Hamiltonian to the orbital-dependent part and the orbital-independent part as $H_{\mathrm{ex1}} = h_1\sigma_{\mathrm{z}}\otimes\tau_{\mathrm{z}}$ and $H_{\mathrm{ex2}} = h_2\sigma_{\mathrm{z}}\otimes I$, respectively,[15, 20] where h_1 and h_2 are the field strength along the z direction, σ_{z} and τ_{z} are Pauli matrices for spin and pseudospin, respectively. If the field only couples to spin ($H_{\mathrm{ex1}} = 0$), the Fermi surface will split into two concentric spheres. If the field couples to spin and orbit both ($H_{\mathrm{ex1}} \neq 0$), the Fermi surface will split into two separate Weyl pockets.[3, 20] Note that in this case, the system is a Weyl semimetal, which does not require the protection of crystal symmetry. Therefore, when considering the hybridization gap Δ induced by magnetic field, there will be a competition between the orbit-coupled field strength h_1 and the field-generated gap term Δ. Since the Landé g factor in Cd_3As_2 is relatively large, it is possible that the Fermi arc surface states are preserved when high field breaks the four-fold symmetry. Such time-reversal-symmetry-breaking effect has been recently observed in Cd_3As_2 through anomalous Nernst effect.[21]

Supplementary References

1. Moll, P. J., *et al.* Transport evidence for Fermi-arc-mediated chirality transfer in the Dirac semimetal Cd_3As_2. *Nature* **535**, 266-270 (2016).
2. Cao, J., *et al.* Landau level splitting in Cd_3As_2 under high magnetic fields. *Nat. Commun.* **6**, 7779 (2015).
3. Liang, T., Gibson, Q., Ali, M. N., Liu, M., Cava, R. J., Ong, N. P. Ultrahigh mobility and giant magnetoresistance in the Dirac semimetal Cd_3As_2. *Nat. Mater.* **14**, 280-284 (2014).
4. Chen, Z.-G., *et al.* Scalable Growth of High Mobility Dirac Semimetal Cd_3As_2 Microbelts. *Nano Lett.* **15**, 5830-5834 (2015).
5. Nicholas, R. J., Haug, R. J., Klitzing, K. v., Weimann, G. Exchange enhancement of the spin splitting in a GaAs-$Ga_xAl_{1-x}As$ heterojunction. *Phys. Rev. B* **37**, 1294-1302 (1988).
6. Zhang, Y., Bulmash, D., Hosur, P., Potter, A. C., Vishwanath, A. Quantum oscillations from generic surface Fermi arcs and bulk chiral modes in Weyl semimetals. *Sci. Rep.* **6**, 23741 (2016).
7. Narayanan, A., *et al.* Linear magnetoresistance caused by mobility fluctuations in the n-doped Cd_3As_2. *Phys. Rev. Lett.* **114**, 117201 (2015).
8. Lv, B., *et al.* Observation of Fermi-Arc Spin Texture in TaAs. *Phys. Rev. Lett.* **115**, 217601 (2015).
9. Xu, S.-Y., *et al.* Spin Polarization and Texture of the Fermi Arcs in the Weyl Fermion Semimetal TaAs. *Phys. Rev. Lett.* **116**, 096801 (2016).
10. Fu, Y. S., Hanaguri, T., Igarashi, K., Kawamura, M., Bahramy, M. S., Sasagawa, T. Observation of Zeeman effect in topological surface state with distinct material dependence. *Nat. Commun.* **7**, 10829 (2016).

11. Potter, A. C., Kimchi, I., Vishwanath, A. Quantum oscillations from surface Fermi arcs in Weyl and Dirac semimetals. *Nat. Commun.* **5**, 5161 (2014).
12. Zhang, Z., *et al.* Zeeman effect of the topological surface states revealed by quantum oscillations up to 91 Tesla. *Phys. Rev. B* **92**, 235402 (2015).
13. Wang, Z., Weng, H., Wu, Q., Dai, X., Fang, Z. Three-dimensional Dirac semimetal and quantum transport in Cd_3As_2. *Phys. Rev. B* **88**, 125427 (2013).
14. Bompadre, S., Biagini, C., Maslov, D., Hebard, A. Unambiguous determination of the g factor for holes in bismuth at high B/T. *Phys. Rev. B* **64**, 073103 (2001).
15. Jeon, S., *et al.* Landau quantization and quasiparticle interference in the three-dimensional Dirac semimetal Cd_3As_2. *Nat. Mater.* **13**, 851-856 (2014).
16. Akrap, A., *et al.* Magneto-Optical Signature of Massless Kane Electrons in Cd_3As_2. *Phys. Rev. Lett.* **117**, 136401 (2016).
17. Kargarian, M., Randeria, M., Lu, Y.-M. Are the surface Fermi arcs in Dirac semimetals topologically protected? *Proc. Natl. Acad. Sci. U.S.A.* **113**, 8648–8652 (2016).
18. Narayan, A., Di Sante, D., Picozzi, S., Sanvito, S. Topological Tuning in Three-Dimensional Dirac Semimetals. *Phys. Rev. Lett.* **113**, 256403 (2014).
19. Analytis, J. G., McDonald, R. D., Riggs, S. C., Chu, J.-H., Boebinger, G., Fisher, I. R. Two-dimensional surface state in the quantum limit of a topological insulator. *Nat. Phys.* **6**, 960-964 (2010).
20. Wang, Z., *et al.* Dirac semimetal and topological phase transitions in A_3Bi (A=Na, K, Rb). *Phys. Rev. B* **85**, 195320 (2012).
21. Liang, T., *et al.* Anomalous Nernst Effect in the Dirac Semimetal Cd_3As_2. *Phys. Rev. Lett.* **118**, 136601 (2017).
